# Stochastic Processes Dominate the Assembly of Soil Bacterial Communities of Land Use Patterns in Lesser Khingan Mountains, Northeast China

**DOI:** 10.3390/life14111407

**Published:** 2024-10-31

**Authors:** Junnan Ding, Shaopeng Yu

**Affiliations:** Heilongjiang Province Key Laboratory of Cold Region Wetland Ecology and Environment Research, Harbin University, Harbin 150086, China; wetlands1972@126.com

**Keywords:** high-throughput sequencing, microbial community, microbial function, soil biodiversity, community assembly processes

## Abstract

To meet the demands of a growing population, natural wetlands are being converted to arable land, significantly impacting soil biodiversity. This study investigated the effects of land use changes on bacterial communities in wetland, arable land, and forest soils in the Lesser Khingan Mountains using Illumina MiSeq 16S rRNA sequencing. Soil physicochemical properties and enzyme activities were measured using standard methods, while microbial diversity was assessed through sequencing analysis. Our findings revealed that forest soils had significantly higher levels of total potassium (2.62 g·kg^−1^), electrical conductivity (8.22 mS·cm^−1^), urease (0.18 mg·g^−1^·d^−1^), and nitrate reductase (0.13 mg·g^−1^·d^−1^), attributed to rich organic matter and active microbial communities. Conversely, arable soils showed lower total potassium (1.94 g·kg^−1^), reduced electrical conductivity, and suppressed enzyme activities due to frequent tilling and fertilization. Wetland soils exhibited the lowest values primarily due to water saturation, which limits organic matter decomposition and microbial activity. Land use changes notably reduced microbial diversity, with conversion from forest to arable land leading to habitat loss. Forest soils supported higher abundances of Proteobacteria (37.59%) and Actinobacteriota (34.73%), while arable soils favored nitrogen-fixing bacteria. Wetlands were characterized by chemoheterotrophic and anaerobic bacteria. Overall, these findings underscore the profound influence of land use on soil microbial communities and their functional roles, highlighting the need for sustainable management practices.

## 1. Introduction

Land use changes influence soil nutrient cycling by modifying environmental conditions such as water and temperature, affecting nutrient transformation and flow, as well as the composition and activity of soil microorganisms [1,2]. These changes can directly or indirectly alter soil microbial communities, impacting their structure and function [3,4]. Rational land use is essential for enhancing soil structure and promoting regional ecological restoration [5]. Microorganisms, as critical biological components of soil, regulate the fixation, decomposition, and mineralization of soil nutrients, which in turn influences plant growth, community structure, and ecosystem dynamics [6].

Soil microbial communities are highly sensitive to environmental changes and are frequently used as indicators of soil quality [7]. Understanding the structure and diversity of these communities is crucial for elucidating interactions between plants, microorganisms, and soil nutrient cycles. Land use patterns significantly affect soil microbial communities by altering physical and chemical soil properties. Variations in farming methods and plant litter input can lead to substantial changes in soil conditions such as moisture and temperature, nutrient availability, and microbial community dynamics [8,9]. Different wetland land use types can impact the functional diversity of microorganisms that utilize various carbon sources, such as carbohydrates and aromatic compounds [10].

Research has shown that during the succession from natural wetlands to cultivated systems, microbial diversity often decreases with increasing carbon inputs [11]. Conversely, fluctuations between wet and dry soil conditions in reclaimed wetlands can enhance microbial diversity. Currently, the impact of land use patterns on soil bacterial and fungal communities remains inconclusive, possibly due to significant geographical variations in the relationship between wetland use patterns, soil physical and chemical environments, and soil microbial communities [12]. Therefore, exploring the effects of land use changes on soil microbial structure and diversity is a prominent topic in global change research [13].

Research on microbial community structure reveals the diversity characteristics present in various habitats. However, a comprehensive understanding of these diversity characteristics necessitates further investigation into the structuring mechanisms [14]. Large-scale studies have identified the involvement of both deterministic and stochastic processes in community construction [15]. While some research indicates that stochastic processes predominate, other studies suggest that deterministic processes are more influential [16]. This discrepancy highlights ongoing debates regarding the mechanisms underlying community construction [17]. The relative significance of these processes varies across different ecosystems, research scales, habitat conditions, and biological attributes [18,19,20]. In the Lesser Khingan Mountains area, wetland land use has been transformed significantly, leading to alterations in soil physical and chemical properties [21]. While existing studies have documented the effects of land use on soil characteristics, there is a notable gap in understanding how these changes specifically influence soil microbial taxa and their functional responses.

Based on the hypothesis that “soil microbial taxa have different responses to soil physical and chemical environmental changes driven by wetland land use patterns”, this study aims to clarify the changing characteristics of soil physical and chemical properties following alterations in wetland land use patterns in the Lesser Khingan Mountains area. Using high-throughput sequencing, the study reveals the structure and diversity characteristics of soil bacterial communities under different land use patterns. Additionally, it analyzes the coupling characteristics between changes in soil physical and chemical properties caused by land use changes and the resulting soil microbial community characteristics. This research provides a scientific basis for understanding the evolution and mechanisms of soil microorganisms in various wetlands in the cold regions of northern China under global change [22,23].

## 2. Materials and Methods

### 2.1. Site Description

**Figure 1:**
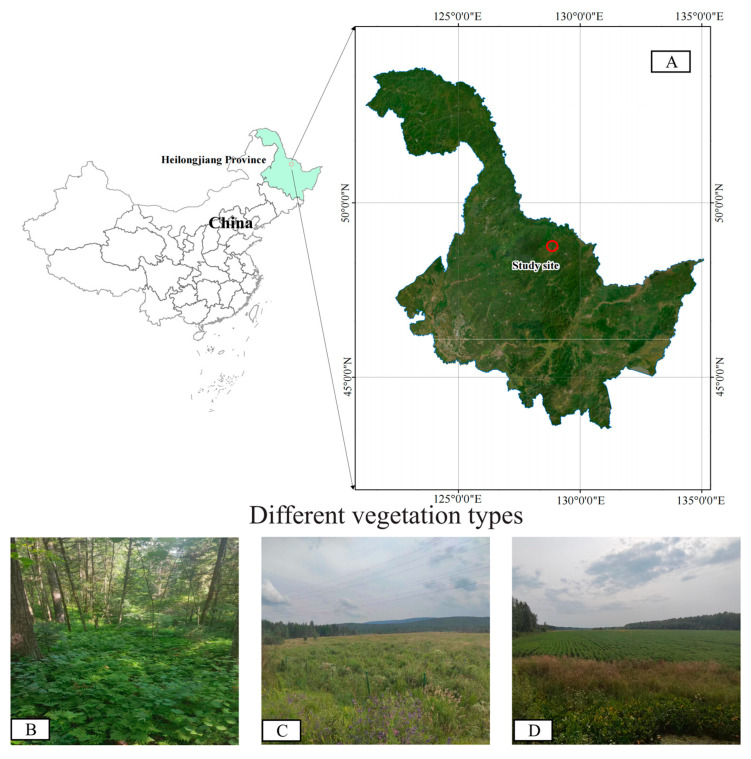
Map showing different land use patterns’ location in the study. (**A**): Test samples’ geographical location; (**B**): forestland (FL); (**C**): arable land (AL); (**D**): wetland (WL).

The study area is located in the Hongxing National Nature Reserve (Figure 1), within the Lesser Khingan Mountains, situated in the northeast of Heilongjiang Province, China (128°21′40″~128°53′30″ E and 48°41′20″~49°11′00″ N). Covering an area of 111,995.3 hectares, the reserve experiences a northern temperate continental monsoon climate, characterized by an annual average temperature of −0.7 °C, an average precipitation range of 500–610 mm, and an average relative humidity of 71.1%. The Hongxing National Nature Reserve is notable for its high biodiversity, encompassing a range of ecosystems including typical swamp, meadow wetland, shrub, and forest vegetation. The reserve is home to 1929 species, among which 885 are plant species and 197 are bryophytes distributed across 49 families. Additionally, the reserve contains 38 species of ferns from 11 families and 650 species of seed plants from 88 families [24]. The soils within the World Reference Base for Soil Resources (WRB) of Sucre are classified as Alfisols, Mollisols, and Vertisols [25]. The wetland park within the reserve is categorized into seven types of wetland: rivers, flood plains, herbs, mosses, swamp meadows, shrub swamps, and forest swamps. The primary landscape of the park is dominated by herb and swamp meadow wetlands, which are surrounded by agricultural lands.

### 2.2. Experimental Design

The vegetation conditions in the soil sampling area are as follows. (1) forestland (FL, formerly degraded wetlands): primary forest with mixed coniferous and broad-leaved forests as the main plant types. (2) Arable land (AL, formerly wetland): the wetland was converted to arable land 32 years ago, and the main planting crop is maize (*Zea mays* L.), which is continuously planted once a year for either silage or grain production. (3) Wetland (WL, formerly arable land): the main dominant plants are *Deyeuxia angustifolia*, *Carex lehmanii*, *Carex meyeriana*, *Carex appendiculata*, *Caltha palustris*, *Cyamus flavicomus*, and *Pedialaris langiflora*. At the end of each April, agricultural machinery tillage is conducted once, and the maize is sown. Farm fertilization and field management are performed according to the local practices. The crop is harvested at the end of September each year, and the land is idle from the end of October to the middle of the following April [26].

### 2.3. Sample Collection

This experiment was conducted from 20 August to 3 September 2022, in the Key Laboratory of Wetland Ecology and Environmental Research of Harbin University (Harbin, China) and the Hongxing National Nature Reserve in Heilongjiang Province, China. According to the characteristics of land use in the nature reserve, three blocks of meadow wetlands, forests, and the surrounding soybean arable land were selected in a 100 × 100 m quadrat. Five sampling points were established according to the “S” type. A 5 cm soil shovel was used to collect soil samples that were 0~0.20 m deep at each sampling point. The mixed soil samples were combined into one replicate, and each sample was collected three times. In each quadrat, after the fresh soil sample was passed through a 2 mm sieve, the root residues were removed and placed in a sampling box. Part of the soil samples were brought back to the laboratory for storage as soon as possible at −20 °C for analysis of the bacterial microbial community structure. Another part was stored at 4 °C to determine the physical and chemical properties of the soil.

### 2.4. Soil Chemical Analysis

A pH meter was used to measure the pH of soil samples. The Walkley–Black titration method was carried out to determine the soil organic carbon (SOC) [27]. The methods of concentrated H_2_SO_4_ digestion and Kjeldahl were used to determine the total nitrogen (TN) content of the soil samples [28]. The content of alkali-hydrolyzable N (AN) in the soil samples was determined using the alkali-hydrolyzable diffusion method [29]. The total content of phosphorus (TP) of the soil samples was determined by HClO_4_ and H_2_SO_4_ digestion molybdenum antimony anticolorimetry. Available phosphorus (AP) in the soil samples was determined by NaHCO_3_ extraction molybdenum antimony anticolorimetry [30]. Total potassium (TK) in the soil samples was determined by digesting soil samples with a mixture of nitric, perchloric, and hydrofluoric acids, followed by flame photometry or atomic absorption spectrophotometry [31]. Available potassium (AK) was extracted using ammonium acetate (1 M, pH 7.0) and quantified using flame photometry or atomic absorption spectrometry [32]. Electrical conductivity (EC) is measured by preparing a 1:5 soil–water suspension and using an EC meter to assess the electrical conductivity of the solution [33]. Urease activity is measured by incubating soil with a urea solution and quantifying the ammonium (NH_4_^+^) released using a colorimetric assay [34]. Soil samples are incubated with casein as a substrate, and the release of amino acids or peptides is measured using spectrophotometry [35]. β-glucosidase activity is determined by incubating soil with p-nitrophenyl-β-D-glucopyranoside and measuring the p-nitrophenol released via colorimetric assay [36]. Measured by incubating soil with nitrate and assessing the amount of nitrite (NO_2_^−^) formed using a colorimetric method with sulfanilamide [37]. Cellulase activity is measured by incubating soil with carboxymethyl cellulose (CMC) as a substrate and quantifying the reducing sugars released, often using the dinitrosalicylic acid (DNS) method [38].

### 2.5. Soil DNA Extraction and High-Throughput Assay

Genomic DNA was extracted from 0.5 g of soil using the Omega E.Z.N.A.^®^ Soil DNA Kit (Omega Bio-Tek, Norcross, GA, USA) following the manufacturer’s protocol. The quality of the extracted DNA was assessed by agarose gel electrophoresis. A two-step polymerase chain reaction (PCR) was performed on a GeneAmp 9700 PCR system (Applied Biosystems, Thermo Fisher Scientific, Waltham, MA, USA). In the first step, universal primers 515F (5′-GTGCCAGCMGCCGCGGTAA-3′) and 907R (5′-CCGTCAATTCMTTTRAGTTT-3′) were used to amplify the V3–V4 region of the bacterial 16S rRNA gene. In the second step, barcodes were added. PCR conditions included an initial denaturation at 95 °C for 3 min, followed by 25 cycles of denaturation at 95 °C for 30 s, annealing at 55 °C for 30 s, and extension at 72 °C for 45 s, with a final extension at 72 °C for 10 min. The PCR product was cleaned using a PCR cleanup kit (Omega Bio-Tek) before quantification with a QuantiFluor^®^-ST fluorometer (Promega, Madison, WI, USA) and adjusted for sequencing. The samples were then sent to Shanghai Meiji Biotechnology Co., Ltd. (Shanghai, China) for high-throughput sequencing on the Illumina HiSeq 2500 PE250 platform (San Diego, CA, USA) [39].

### 2.6. Statistical Analysis

We performed quality control on the sequencing data, typically setting a quality score threshold of Q20 (i.e., an error rate of less than 1% per base) and removing sequences shorter than 200 bp. Using QIIME (Caporaso Lab, Northern Arizona University) and DADA2 (Benjamin Callahan, North Carolina State University), we filtered out sequences containing ambiguous bases (N) and low-quality sequences, ensuring that the final sequence purity exceeded 90%. Community diversity parameters (Shannon and Simpson indices) and community richness parameters (ACE and Chao indices) were used to conduct alpha diversity analyses with mothur software [40]. The R software package (version vegan) was used to analyze and describe the β calculation visually. Diversity was examined by one-way analysis of variance (ANOVA) and the least significant difference. Data were statistically analyzed using Microsoft Excel 2007 (Redmond, WA, USA) and SPSS 22.0 (IBM, Inc., Armonk, NY, USA). Microbial functions of the soil bacteria were predicted by FAPROTAX and FUN Guide [41,42]. For the functional gene composition prediction using PICRUSt2, 70% of the OTUs with functional gene annotations were utilized, with unclassified OTUs excluded to ensure the robustness of functional predictions [43]. While a portion of the microbial diversity remains unclassified, the data used represent more than 50% of the identified bacterial communities in our study. We believe this is sufficient to make reliable predictions, but we acknowledge that the exclusion of unidentified bacteria may affect the completeness of the functional analysis [44].

Community construction analysis refers to the method based on phylogenetic diversity proposed by Stegen et al. to calculate the βNTI value of community construction under different ecosystem types [45,46]. When |βNTI| < 2, it indicates that random processes dominate microbial community construction; when |βNTI| > 2, it indicates that deterministic processes dominate microbial community construction. The normalized stochastic ratio (NST) assesses the relative significance of stochastic mechanisms in the assembly of soil bacterial communities, utilizing a threshold of 50% to identify whether deterministic processes or stochastic processes are more dominant [47]. For the NCM, βNTI, and NST analyses, 70% of the OTUs were successfully identified and included, while the remaining 30% (unclassified or unknown OTUs) were excluded [48]. RCbray was further calculated to quantify the effects of selection, restricted dispersal, uniform dispersal, and drift on community changes. BugBase was used to annotate the functions of bacteria, and the OTU table that clustered by 97% sequence similarity was used as the input file. The OTU table was standardized by the predicted number of 16S copies, and the microbial phenotype was then predicted using the preprocessed database. The threshold was automatically selected by the BugBase tools. The samples were divided into the following seven categories: aerobic, anaerobic, stress tolerant, Gram negative, Gram positive, and potentially pathogenic [49,50].

## 3. Results

### 3.1. Soil Chemical Properties and Soil Enzymes

Soil properties and soil enzymes responded differently to the presence of land use patterns as shown in Table 1. The pH of soil samples was relatively neutral. Total potassium, electrical conductivity, urease, and nitrate reductase of the FL were significantly higher than those of the AL and WL (*p* < 0.05), and the contents of SOC of the wetland were 15.27 and 20.42% lower than those of the forestland and farmland, respectively. Total nitrogen and available potassium were not statistically significantly different between all soil samples. β-glucosidase, protease, and cellulase of the wetland were significantly higher than those of the arable land and forestland (*p* < 0.05).

### 3.2. Effects of Land Use Patterns on Microbial Alpha Diversity

The alpha diversity index quantifies the number and relative abundance of species within a community, reflecting interactions such as competition or symbiosis among species sharing the same habitat. An analysis of the diversity abundance of bacterial and microbial species reveals changes in the alpha diversity index, as indicated by statistical *t*-tests presented in Table 2. While the Shannon index for soil bacteria did not show significant differences across the three land use types (Table 2), The patterns of the ACE index exhibited variation among these land use types; however, the differences between forestland, arable land, and wetland were not statistically significant.

### 3.3. Analysis of Soil Bacterial Structure in Different Land Use Patterns

**Figure 2:**
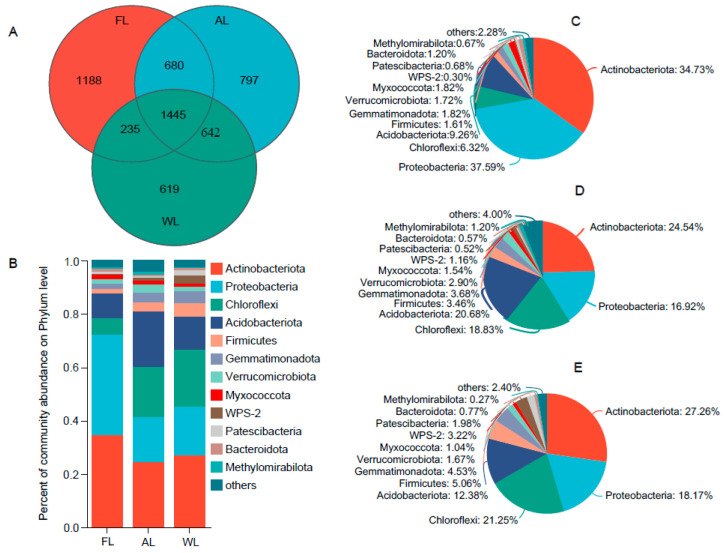
Relative abundance of soil bacterial communities under different land uses at the phylum level. Abbreviations, FL, forestland; AL, arable land; WL, wetland. (**A**) Venn diagram of soil microorganisms based on the level of OTUs. (**B**) Relative abundance of soil bacterial communities under different land uses at the phylum level. (**C**) Community analysis pieplot on phylum level in FL. (**D**) Community analysis pieplot on phylum level in AL. (**E**) Community analysis pieplot on phylum level in WL.

The intersection of OTU sequences exhibiting over 97% similarity with the soil bacterial communities associated with each treatment is depicted as a Venn diagram (Figure 2). A total of 10,035 operational taxonomic units (OTUs) classified into 41 bacterial phyla were identified in the analyzed soil (Figure 2A). The abundance of bacterial OTUs was ranked as follows: AL (3564) > FL (3548) > WL (2941). Among these, 1188 OTUs were exclusively found in forestland soil bacteria, representing 11.83% of the overall OTU sequences identified (Figure 2A). Figure 1B illustrates that while the composition of the predominant bacterial species was comparable across different soil samples, the abundance levels of bacterial communities varied significantly among the land use patterns. At the taxonomic classification level for the examined soil samples, the dominant bacterial groups were Actinobacteriota, Proteobacteria, Chloroflexi, and Acidobacteriota. The relative proportions of Proteobacteria and Actinobacteriota were greatest in the forestland soil (Figure 2C). Similarly, the relative abundance of Actinobacteriota, Acidobacteriota, Chloroflexi, and Proteobacteria was notably high in arable land (Figure 2B); in the wetland, the highest relative abundance was recorded for Actinobacteriota and Chloroflexi (Figure 2C).

### 3.4. Correlation Analysis of Environmental Factors

**Figure 3:**
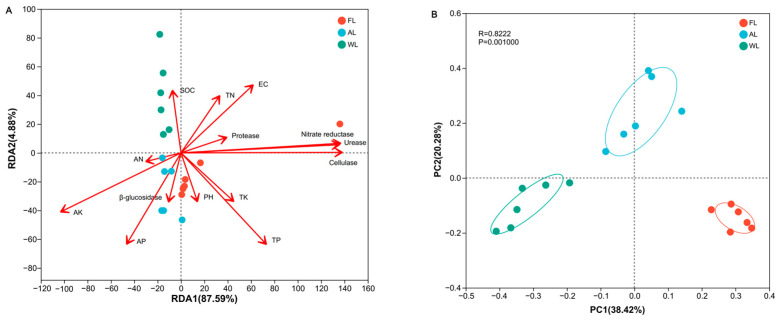
Environmental factor correlation analysis. The RDA (**A**) and PCoA diagram (**B**) of the soil bacterial community at the OTU level. Abbreviations, SOC, soil organic carbon; TN, total nitrogen; TP, total phosphorus; TK, total potassium; AN, available nitrogen; AP, available phosphorus; AK, available potassium; EC, electrical conductivity. FL, forestland; AL, arable land; WL, wetland.

Redundancy analysis (RDA) of the bacterial data revealed that soil physicochemical properties explained the majority of the variance in the RDA results as the first two axes explained 87.59% (Figure 3A). The wetland bacterial communities were positively correlated with SOC, while the AL was positively correlated with AP, AK, AN and β-glucosidase. The FL was positively correlated with soil pH, TK, TP, nitrate reductase, urease, and cellulase. In the analysis of beta diversity, WL was separated from the other two land use types, suggesting a clear distinction in the bacterial community structures between wetlands and forests and arable land (Figure 3B). In general, the PCoA explained 58.7% of the variation in the bacterial communities.

**Figure 4:**
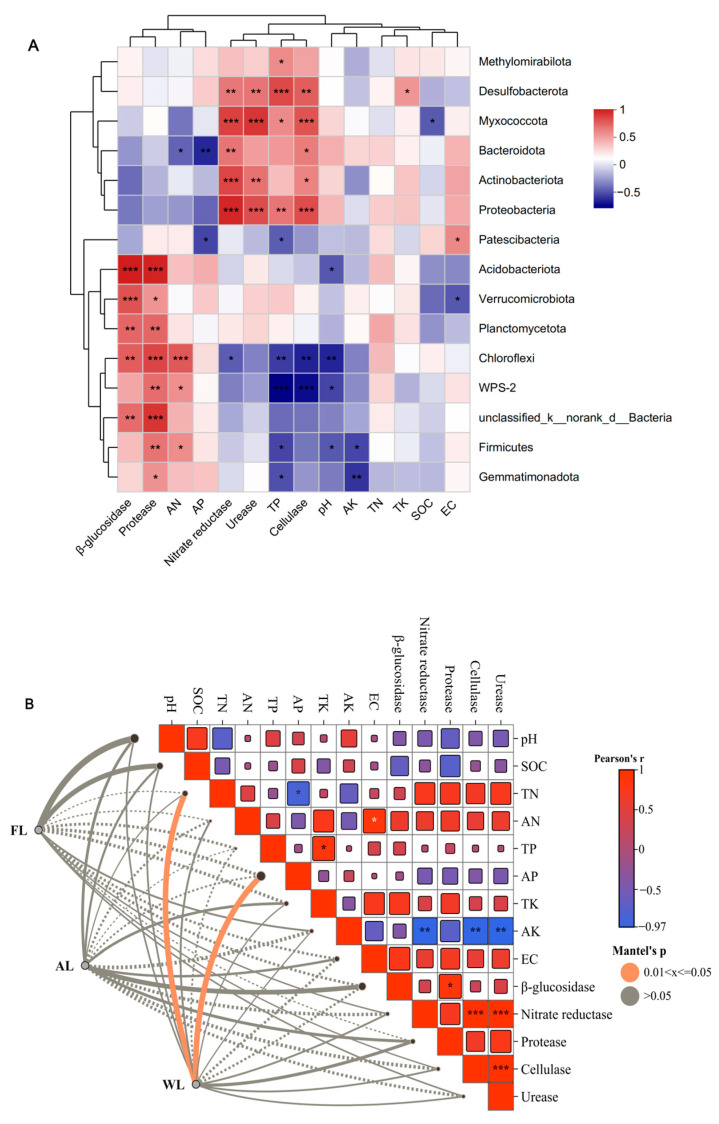
Correlation heat map of the top fifteen phyla and soil properties and soil enzymes (**A**). Mantel analysis on the relationships between different land uses patterns and oil properties and soil enzymes (**B**). X and Y axes are environmental factors and phyla. The legend showed the color range of different R values. Significant values are shown as: * *p* < 0.05; ** *p* < 0.01; *** *p* < 0.001. Abbreviations, SOC, soil organic carbon; TN, total nitrogen; TP, total phosphorus; TK, total potassium; AN, available nitrogen; AP, available phosphorus; AK, available potassium; EC, electrical conductivity. FL, forestland; AL, arable land; WL, wetland.

The heatmap of the correlation between soil environmental factors, soil enzymes, and soil microbial community composition (Figure 4A) indicates varying degrees of correlation between these factors at the phylum level. Specifically, AN shows a significant positive correlation with Chloroflexi, WPS-2, and Firmicutes and a significant negative correlation with Bacteroidota. AP is significantly negatively correlated with Bacteroidota and Patescibacteria. TP exhibits a significant positive correlation with Desulfobacterota, Proteobacteria, Methylomirabilota, and Myxococcota and a significant negative correlation with WPS-2, Patescibacteria, Chloroflexi, Firmicutes, and Gemmatimonadota. Soil pH is significantly negatively correlated with Chloroflexi, WPS-2, Acidobacteriota, and Firmicutes. AK is significantly negatively correlated with Firmicutes and Gemmatimonadota. TK shows a significant positive correlation with Desulfobacterota (*p* < 0.05). SOC is significantly negatively correlated with Myxococcota (*p* < 0.05). EC is significantly positively correlated with Patescibacteria (*p* < 0.05) and significantly negatively correlated with Verrucomicrobiota (*p* < 0.05). β-glucosidase is highly significantly positively correlated with Acidobacteriota and Verrucomicrobiota (*p* < 0.001). Protease is highly significantly positively correlated with Acidobacteriota, Chloroflexi, and unclassified_k_norank_d_Bacteria (*p* < 0.001). Nitrate reductase is highly significantly positively correlated with Myxococcota, Actinobacteriota, and Proteobacteria (*p* < 0.001) and significantly negatively correlated with Chloroflexi (*p* < 0.05). Urease is significantly positively correlated with Myxococcota, Proteobacteria, Desulfobacterota, and Actinobacteriota. Cellulase is highly significantly positively correlated with Proteobacteria and Myxococcota and significantly negatively correlated with WPS-2 and Chloroflexi. As shown in Figure 4B, WL exhibits a strong positive correlation with soil properties such as SOC, TN, and AN, as well as with enzyme activities like protease and cellulase. Additionally, AL and FL show a significant correlation between EC and AK.

### 3.5. The Construction Process of Bacterial Communities Under Different Land Use Patterns

**Figure 5:**
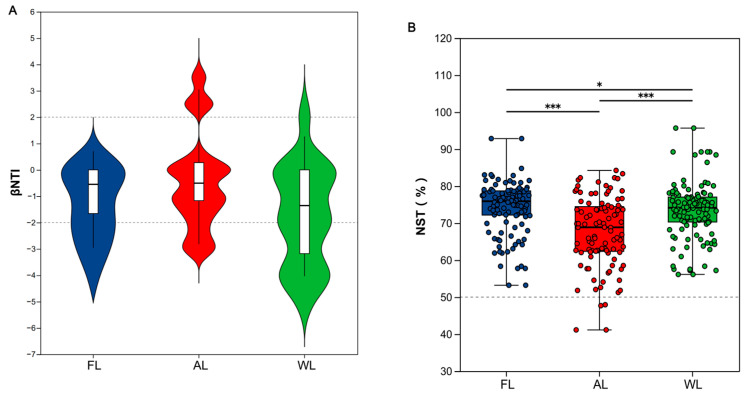
βNTI values (**A**) and normalized stochasticity ratio (NST, (**B**)) of the soil bacterial community under different land use patterns (* *p*< 0.05, *** *p*< 0.001). Abbreviations, FL, forestland; AL, arable land; WL, wetland.

As illustrated in Figure 5, both deterministic and stochastic processes play a role in the construction of soil bacterial communities. The βNTI values for soil bacterial community assembly across various land use patterns were calculated. The results (Figure 5A) indicated that |βNTI| < 2, suggesting that at this local scale, random processes predominantly influenced the bacterial community construction across different land use patterns. Additionally, we computed normalized stochastic ratios (NST) to quantify the contributions of deterministic and stochastic processes among soil bacteria under varying land uses (Figure 5B). The NST values for bacterial communities in FL, AL, and WL soils exceeded the 50% threshold, with an average of 71.89%, indicating that the assembly of soil bacterial communities in the study area is primarily driven by random processes. Furthermore, the NST values for both FL and WL land use modes were significantly higher than those for AL (*p* < 0.001).

### 3.6. Analysis of Changes in Microbial Community Assembly Ecological Processes

**Figure 6:**
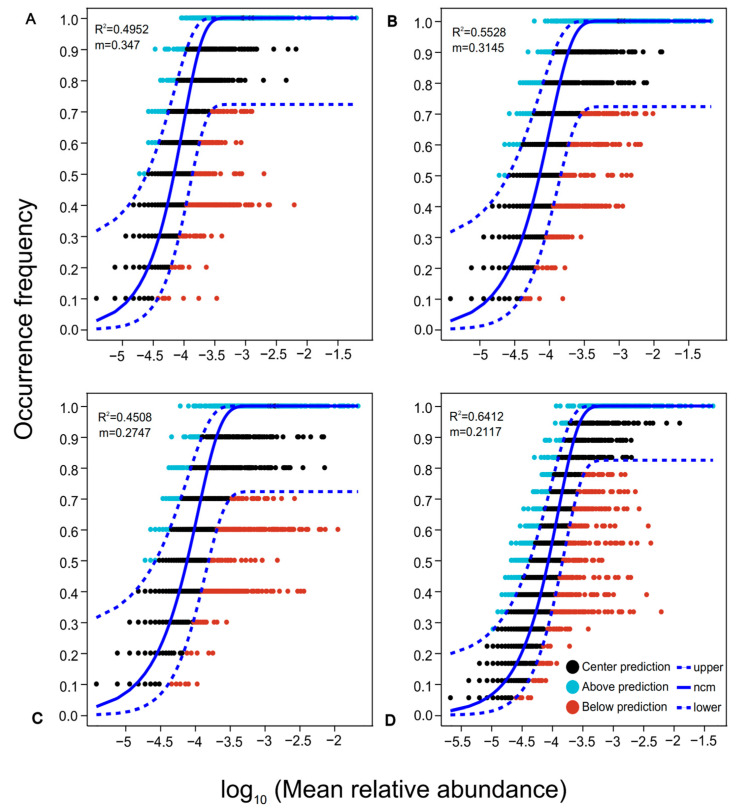
Neutral community models under different ecosystems. (**A**): Forestland (FL). (**B**): Arable land (AL). (**C**): Wetland (WL). (**D**): Overall level. The solid line indicates the fitting of the neutral model, while the upper and lower dotted lines represent the 95% confidence level of the model prediction. Blue represents OTUs above the confidence interval, black represents OTUs within the confidence interval, and red represents OTUs below the confidence interval.

The neutral community model (NCM) quantitatively analyzes the stochastic processes involved in community construction (Figure 6). The results indicate that the model accounts for 49.52%, 55.28%, and 45.08% of the community variation in forest, arable land, and wetland ecosystems, respectively, and overall explains 64.12%. The m value quantifies the migration rate at the community level, with values in forestland, arable land, and wetland ecosystems recorded at 0.0347, 0.3145, and 0.2747, respectively. All m values are significantly less than 1, further confirming that random processes predominantly influence the construction of soil bacterial microbial communities across different land uses patterns.

**Figure 7:**
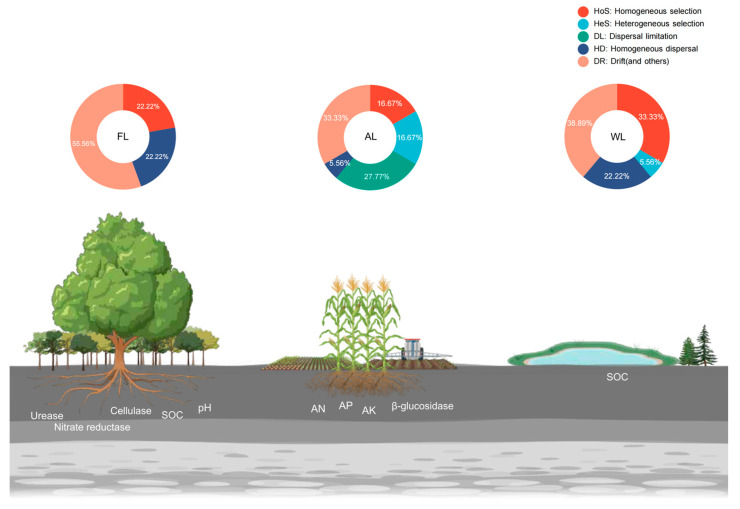
Concept figure of the assembly of soil microbial community under different land use types. Abbreviations, FL, forestland; AL, arable land; WL, wetland; SOC, soil organic carbon; AN, available nitrogen; AP, available phosphorus; AK, available potassium.

RCbray was further calculated to distinguish the relative contributions of dispersal limitation, drift, uniform dispersal, and selection to community changes. The results (Figure 7) indicate that the relative roles of deterministic and stochastic processes vary across different land use patterns. In the construction of bacterial communities, the homogeneous selection was recorded as 33.33%, 16.67%, and 22.22% in WL, AL, and FL, respectively. In contrast, the random processes were averaged and ranked, revealing that homogeneous dispersal variation accounted for 50% and uniform dispersal for 0.37%. Additionally, the effect of dispersal limitation in AL was the weakest, at 27.78%, while the drift effect was the strongest, at 33.33%.

### 3.7. Changes in the Microbial Functions of Soil Bacteria

**Figure 8:**
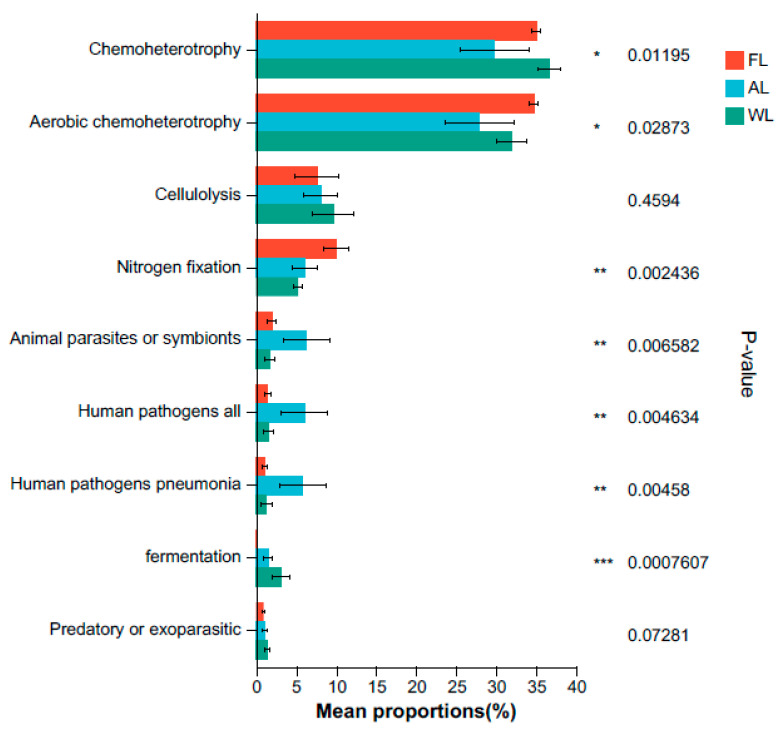
Abundance of soil bacterial community predicted by functional gene composition. * *p* < 0.05; ** *p* < 0.01; and *** *p* < 0.001. Abbreviations, FL, forestland; AL, arable land; WL, wetland.

The FAPROTAX algorithm selected the top nine functional groups in the soil bacterial community according to their sequence ranking and statistically analyzed the functional bacterial abundance. As shown in Figure 8, land use patterns can change the abundance of soil functional microorganisms. The relative abundance of chemoheterotrophic bacteria was highest in WL (36.65%), followed closely by FL (35.01%) and AL (29.81%), with the abundance in FL and AL being 18.65% and 4.47% lower than that in WL, respectively (*p* < 0.05). The relative abundance of aerobic chemoheterotrophic bacteria was recorded at 34.73% in FL and 31.96% in WL. Furthermore, the relative abundance of nitrogen fixation bacteria was notably higher in AL (10.02%), which represented increases of 39.59% and 47.63% compared to WL and FW, respectively (*p* < 0.01). Similarly, the levels of animal parasites or symbionts, all human pathogens, and human pneumonia pathogens were found to be higher in AL than in FL and WL (*p* < 0.01).

### 3.8. BugBase Phenotype Prediction

**Figure 9:**
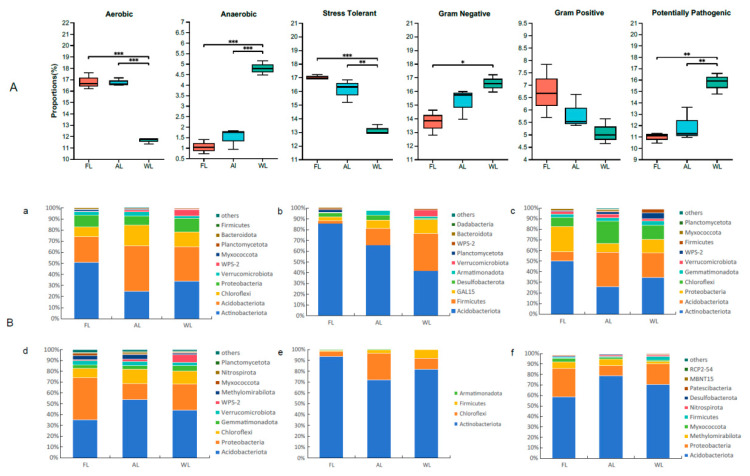
BugBase phenotype prediction of soil bacterial communities in different land use patterns. * *p* < 0.05; ** *p* < 0.01; and *** *p* < 0.001. (**A**) Using Bugbase in different land use patterns. (**B**) Species and phenotype contribution analysis. (**a**) Aerobic. (**b**) Anaerobic. (**c**) Stress tolerant. (**d**) Gram negative. (**e**) Gram positive. (**f**) Potentially pathogenic. Abbreviations, FL, forestland; AL, arable land; WL, wetland.

The phenotypic abundance analysis of six types of bacteria (aerobic, anaerobic, stress tolerant, Gram negative, Gram positive, and potentially pathogenic) predicted by BugBase based on different land use patterns is presented in Figure 9. The land use patterns of FL and AL resulted in an increase in the relative abundance of bacterial communities exhibiting aerobic, stress-tolerant, and Gram-positive functional phenotypes compared to WL, whereas the functional phenotypes of anaerobic, Gram-negative, and potentially pathogenic bacteria were significantly reduced. At the microbial community level, the relative abundance of aerobic functional phenotypes in FL and AL soil samples was significantly higher than that in WL (*p* < 0.001). Changes in bacterial relative abundance were primarily driven by Actinobacteriota, Acidobacteriota, and Chloroflexi, which were ultimately influenced by variations in the relative abundance of Proteobacteria, Verrucomicrobiota, WPS-2, Myxococcota, Planctomycetota, Bacteroidota, and Firmicutes. Conversely, the relative abundance of anaerobic bacteria in FL and AL soil samples was significantly lower than that in WL (*p* < 0.001), with Acidobacteriota and Firmicutes being the main drivers of this trend. Notably, the relative abundance of Firmicutes in FL was higher than in AL, while WL experienced decreases of 81.88% and 90.16%, respectively. These changes can be attributed to shifts in GAL15, Desulfobacterota, Armatimonadota, Verrucomicrobiota, and Planctomycetota. The bacterial abundance of the stress-tolerant functional phenotype in FL and AL was higher than that in WL, with changes in bacterial abundance primarily driven by Chloroflexi, Gemmatimonadota, and Verrucomicrobiota. Notably, the relative abundance of Gram-negative bacteria in WL soil samples was significantly greater than that in FL (*p* < 0.05). The bacterial species composition was predominantly dominated by Acidobacteriota, Proteobacteria, and Chloroflexi. Additionally, the changes in bacterial abundance were chiefly influenced by Gemmatimonadota and Verrucomicrobiota. While Gram-positive bacterial abundance in FL and AL soil samples was slightly higher than that in WL, the difference between these groups was not statistically significant. The observed changes in bacterial abundance were primarily attributed to Proteobacteria, Acidobacteria, Actinobacteria, Chloroflexi, and Nitrospirillum. The abundance of Gram-negative bacteria in forest soil samples was found to be higher than in wetland and farmland samples, with a highly significant relationship observed (*p* < 0.01). The variations in bacterial abundance were primarily attributed to Actinobacteriota and Chloroflexi. Additionally, the abundance of potentially pathogenic bacteria in wetland soil samples was significantly greater than that in farmland and agricultural land samples (*p* < 0.01), with the changes in bacterial abundance mainly driven by Acidobacteriota and Proteobacteria.

## 4. Discussion

### 4.1. Effect of Land Use on Soil Bacterial Community Diversity

This study explored the impact of different land use types on TK, EC, urease, and nitrate reductase properties in the Lesser Khingan Mountains region. The results indicated that forestland soils exhibited significantly higher levels of TK, EC, urease, and nitrate reductase compared to arable land and wetland soils (Table 1). This advantage in forestland soils can be attributed to the abundant organic matter and active microbial communities within these ecosystems. The accumulation of plant litter and organic matter decomposition in forests provides a stable source of potassium, enhancing soil electrical conductivity. These processes also support higher activities of urease and nitrate reductase, highlighting the critical role of forestland soils in nutrient and mineral cycling [51,52].

In contrast, agricultural soils showed lower TK levels, which can be linked to frequent tilling and fertilizer application. The nutrient cycling in arable land soils primarily relies on external fertilization. Frequent tilling and harvesting disrupt soil structure, reduce organic matter accumulation, and limit potassium release [53]. Although fertilization increases ion concentrations in soils, frequent irrigation and rainfall result in leaching of salts, thus lowering EC. The urease activity in arable land soils is also suppressed by fertilizer use, and the impact of tilling and harvesting further affects enzyme activity. Wetland soils exhibit even lower levels of TK, EC, urease, and nitrate reductase activity, mainly due to water saturation conditions that limit organic matter decomposition and microbial activity [54,55]. Excessive moisture in wetland soils dilutes soil salts, leading to lower EC, while water-saturated conditions limit oxygen supply, thereby inhibiting microbial metabolism and enzyme activity [56]. The anaerobic environment in wetlands also impacts urease and nitrate reductase activity, reducing their role in nitrogen cycling [57]. These results indicate that wetland soils are less effective in processing nitrogen and minerals compared to forest soils, closely related to their unique hydrological and environmental conditions.

When land use changes, the types of vegetation and the physical and chemical properties of the soil also change, which in turn has a significant impact on soil microbial diversity [58]. Previous research by the authors on the impact of cultivation on the diversity of soil bacterial communities in marsh wetlands found that cultivation can significantly increase the soil bacterial diversity index in marsh wetlands [59]. In this study, the conversion of forestland to arable land resulted in habitat loss and fragmentation, which are some of the main reasons for the decline in biodiversity. Logging and surface disturbances in forest ecosystems lead to a reduction in the living space and food resources for many plant and animal species. Studies have shown that habitat fragmentation not only directly reduces species numbers but also weakens ecosystem functions [60].

Arable land usually undergoes deep plowing, fertilization, and irrigation. While these practices help increase crop yields, they disrupt soil structure, reduce organic matter content, and thus affect the diversity and abundance of soil microbial communities [61]. Secondly, the use of pesticides and fertilizers has a significant impact on the microbial communities in soil and water. The widespread use of pesticides and fertilizers exerts toxic effects on microbial communities, reducing the variety and number of microorganisms, thereby leading to a decrease in the Simpson index, ACE index, and Chao1 index (Table 2). Recent studies have shown that the long-term use of pesticides and fertilizers can reduce beneficial microorganisms in the soil, increase the proportion of harmful microorganisms, and thus disrupt the balance of the ecosystem [62]. Additionally, monoculture is another important factor affecting biodiversity. Farmland typically grows single crops, and this single-ecosystem structure cannot support a diverse range of biological communities, resulting in decreased biodiversity. Research has found that diverse plant communities can support more microbial and animal species, thereby enhancing the stability and function of ecosystems [63]. Therefore, farmland with monoculture has a significantly lower biodiversity index compared to diverse forests.

When arable land is converted into wetlands, the Shannon index, Simpson index, and Chao1 index do not show significant differences, whereas the ACE index decreases. This phenomenon reflects the complex relationships between different ecosystems. Firstly, the restoration capacity of wetlands is an important factor affecting changes in biodiversity. Wetland ecosystems have strong restorative capabilities, and during the conversion of arable land to wetlands, many plant and animal species can quickly recover, maintaining relatively stable Shannon and Simpson indices [64]. This indicates that the overall level of biodiversity in wetland ecosystems does not significantly change during restoration. Secondly, changes in environmental conditions in wetlands are also key factors affecting biodiversity indices. The hydrological conditions, soil moisture, and organic matter content in wetlands influence the growth and distribution of microorganisms and plants. Although overall biodiversity (reflected in the Shannon and Simpson indices) may not change significantly (Table 2), the ACE index may decrease significantly. This is because during the conversion of farmland to wetlands, certain species may not be able to adapt to the new environmental conditions, leading to a decrease in abundance [65]. Furthermore, the presence of wetland-specific species is another important reason for the significant decrease in the ACE index. Wetlands have unique ecological conditions, and the species adapted to these conditions may differ from those in arable land. Therefore, although the overall value of the Chao1 index may not change significantly, changes in the abundance of wetland-specific species can lead to decrease in the ACE index. Studies have shown that wetland-specific species are very sensitive to environmental changes, and significant changes in their abundance can directly impact biodiversity indices [66].

### 4.2. Impacts of Land Use Patterns on Soil Bacterial Community Structure

The impact of different land use types on soil microbial composition is primarily reflected in changes in soil environmental conditions. The differences in soil microbial communities among forest, wetland, and arable land reveal the profound effects of land management practices on soil ecosystems. These differences not only affect the microbial community structure but may also have significant implications for soil health and ecosystem functions [67]. Shifts from forest to cropland or towards agroforestry can significantly influence microbial processes, particularly those related to nutrient cycling and soil health. For instance, the transition to cropland often leads to a reduction in soil organic matter and biodiversity, which can diminish microbial activity and alter community composition [68]. Conversely, agroforestry practices, which integrate trees with crops, can enhance soil microbial diversity and activity by providing additional organic inputs and improving soil structure. These changes can subsequently affect key microbial processes, such as decomposition and nutrient mineralization, ultimately influencing ecosystem functioning. In this study, the abundance of Actinobacteriota and Proteobacteria was significantly higher in forest soils compared to other phyla. This phenomenon is closely related to the high organic matter content and relatively stable environmental conditions of forest soils (Figure 1B) [69]. Forest soils are typically rich in plant residues and organic matter, providing abundant nutritional resources for Actinobacteriota, thereby supporting their high abundance in these soils [70]. Actinobacteriota play a crucial role in decomposing organic matter and promoting nutrient cycling, as they are highly efficient in breaking down complex organic compounds and releasing usable nutrients. Proteobacteria also play a key role in nutrient transformation and microbial interactions in the soil, adapting to variable environmental conditions and forming complex microbial networks. The stability and minimal human intervention in forest soils further support the proliferation of these microorganisms [71]. In contrast, the microbial communities in arable land soils are significantly influenced by agricultural activities such as fertilization and tillage [72]. Fertilization increases the nutrient content of the soil, which in turn promotes the abundance of Actinobacteriota, Acidobacteriota, Chloroflexi, and Proteobacteria [73]. Fertilizers provide ample nitrogen, phosphorus, and potassium, supporting the growth and proliferation of specific microbial groups. For instance, fertilization can lead to soil acidification, which may alter the microbial community structure and function. Tillage disrupts soil structure and modifies soil aeration, affecting microbial growth and activity. These changes in arable soils may lead to reduced microbial diversity and impact soil ecosystem functions. Wetland soils exhibit significantly different microbial communities compared to forestland and arable land soils, primarily due to high-moisture and low-oxygen conditions [74]. In wetland soils, Actinobacteriota and Chloroflexi are more abundant. The high-moisture and low-oxygen conditions in wetlands promote the proliferation of microorganisms adapted to these conditions. The slower decomposition rate of organic matter in wetland soils may lead to increased abundance of Actinobacteriota and Chloroflexi in these environments. The high-moisture and low-oxygen environment affects microbial metabolic processes, allowing anaerobic microorganisms to thrive in wetland soils. The unique environmental conditions in wetlands result in distinct functional and structural characteristics of microbial communities, which are crucial for the health and stability of wetland ecosystems. The mechanisms driving shifts in bacterial communities in response to land use change are critical for understanding ecosystem dynamics and microbial resilience. These shifts can be influenced by factors such as changes in soil pH, nutrient availability, and organic matter inputs [75]. For example, alterations in land use can lead to changes in microbial habitats and resource competition, which directly affect community composition and function [76]. Understanding these mechanisms not only clarifies how bacterial communities respond to environmental changes but also informs land management practices aimed at preserving microbial diversity and ecosystem health.

The bacterial communities in wetland soils are significantly positively correlated with SOC (Figure 2A). The high organic matter content and favorable moisture conditions in wetland ecosystems offer bacteria abundant nutrients and optimal environments for growth. These conditions not only support microbial metabolism but also enhance bacterial diversity and population abundance, contributing to a more dynamic and resilient microbial community [77]. Soil organic carbon, as a major carbon source in wetland soils, not only directly affects bacterial metabolic activities but also indirectly influences bacterial community structures by altering the soil’s physical and chemical properties [78]. The accumulation of organic carbon contributes to the carbon cycle in wetland ecosystems and, by enhancing soil water retention and nutrient supply, further supports microbial growth [79]. Additionally, high activities of protease and cellulase indicate active decomposition of organic matter and nutrient cycling in wetland soils, which help maintain the health and stability of wetland ecosystems [80].

Intensive agricultural activities, such as fertilization and tillage, significantly alter the distribution of soil nutrients and biochemical processes [81,82]. Fertilization increases the nutrient content in the soil, directly impacting bacterial growth and metabolic activities. Nitrogen supply significantly affects the composition and function of soil bacterial communities [83]. Chloroflexi, a widely present soil bacterium, typically dominates environments rich in organic matter. Increased nitrogen provides ample nutrients for Chloroflexi, promoting its growth and metabolic activities. Enhanced nitrogen supply may improve nitrogen cycling efficiency in the soil, strengthening Chloroflexi’s role in nitrogen cycling. This bacterium’s ability to degrade organic matter gives it a significant ecological advantage in nitrogen-rich soils [84]. WPS-2 is an emerging group of bacteria recently discovered in environmental microbiology research, whose ecological functions are not yet fully understood. However, studies have shown that these bacteria have higher abundance in nitrogen-rich environments, likely due to their unique nitrogen metabolism capabilities. WPS-2 may utilize nitrogen as a key nutrient, thereby exhibiting significant growth advantages in nitrogen-rich soils [85]. Firmicutes play an important role in soil ecosystems, especially in the decomposition of organic matter and nitrogen cycling. Increased nitrogen promotes the growth of Firmicutes, possibly because these bacteria can efficiently use nitrogen compounds as metabolic substrates, thereby increasing their competitiveness in nitrogen-rich environments (Figure 4B). Certain species within Firmicutes, such as Bacillus, have been widely studied for their beneficial functions in agricultural soils, including nitrogen fixation and phosphate solubilization [86]. Bacteroidota is commonly found in environments rich in organic matter. However, our study found a significant negative correlation between AN and Bacteroidota (Figure 3A), suggesting that high-nitrogen conditions may be detrimental to the growth of these bacteria. This may be due to high-nitrogen environments altering the microbial ecological balance of the soil, inhibiting the metabolic activities and growth of Bacteroidota. Bacteroidota typically rely on the decomposition of complex organic matter, and high-nitrogen conditions may cause an imbalance in the carbon to nitrogen ratio, affecting their growth [87]. Acidobacteriota and Verrucomicrobiota produce and utilize enzymes, further promoting the carbon cycling process in the soil [88]. Desulfobacterota are sulfate-reducing bacteria that typically dominate in environments rich in organic matter and phosphorus. The increase in total phosphorus likely provides essential nutrients for these bacteria, promoting their growth and metabolic activity (Figure 4B) [89]. Both soil pH and total phosphorus content significantly influence the composition and distribution of soil bacterial communities (Figure 4B). Neutral soil pH is unfavorable for the growth of Chloroflexi, WPS-2, Acidobacteriota, and Firmicutes, whereas a high-phosphorus environment promotes the growth of Desulfobacterota, Proteobacteria, Methylomirabilota, and Myxococcota, while inhibiting the growth of WPS-2, Patescibacteria, Chloroflexi, Firmicutes, and Gemmatimonadota (Figure 4B) [90,91].

### 4.3. Bacterial Community Assembly and Neutral Community Model Analysis

Research has shown that the assembly of soil microbial communities is driven by both stochastic and deterministic processes [92]. Investigating the relative contributions of these processes to community assembly has long been a central challenge in microbial ecology [93]. In this study, the mechanisms of soil bacterial community assembly under different land use types at a local scale were explored, revealing that both deterministic and stochastic processes jointly drive microbial community assembly (Figure 4). Deterministic processes (environmental filtering) play a role in shaping soil microbial communities. The correlation between soil physicochemical properties and bacterial communities shows that certain environmental factors are significantly associated with bacterial communities, indicating that environmental filtering contributes to microbial community assembly in this study area. However, the role of environmental filtering (deterministic processes) appears to be relatively minor, as indicated by |βNTI| < 2. It was found that environmental selection is strongest in forest ecosystems, likely due to the formation of complex but specialized symbiotic networks in forests, which are highly selective due to heterogeneous litter inputs [94]. Similar conclusions have been drawn by other researchers in different mediums, confirming that deterministic processes are involved in the assembly of bacterial communities [95,96]. On the other hand, stochastic processes play a significant role in microbial community assembly. The |βNTI| values for different land use types were all less than 2, suggesting that stochastic processes dominate bacterial community assembly in this study area. This dominance can be attributed to several factors: (1) at the scale of this study, the similarity of bacterial communities decreases significantly with increasing geographical distance, exhibiting a clear distance-decay pattern. (2) The value in the neutral model is much less than 1, indicating that dispersal limitation (a stochastic process) plays a key role in bacterial community assembly (Figure 5). (3) Only a few measured environmental factors showed significant correlations with bacterial communities, suggesting that selective pressures might be relatively weak in this study area. The study found that the diffusion restriction effect is the weakest, while the drift effect is the strongest in the arable land ecosystem. This observation may be attributed to several factors: (1) the strength of biological interactions. Research has demonstrated that biotic interactions are more significant in community construction than abiotic factors. The arable land ecosystem experiences a moderate level of disturbance, which facilitates increased interactions among microbial taxa [97].

### 4.4. Impact of Different Land Use Types on Soil Microbial Function

Soil microbial functions are crucial for ecosystem health and sustainability, encompassing processes such as nutrient cycling, organic matter decomposition, and soil structure formation. Different land use types can significantly impact these microbial functions, leading to variations in soil health, fertility, and overall ecosystem functionality [98]. According to the results, there are significant differences in the relative abundance of chemoheterotrophic bacteria under different land use patterns (Figure 6). This variation can be explained by the unique environmental conditions of wetlands. Wetlands typically have abundant organic matter and high moisture levels, creating an environment that is highly conducive to the growth of chemoheterotrophic bacteria [99]. Small doses of mineral nitrogen (Nmin) fertilizer (20–30 kg N·ha^−1^) applied to leguminous crops provide an initial supply of nitrogen to support early plant growth [100,101]. However, higher doses of mineral nitrogen can inhibit the activity of nodule bacteria, potentially reducing their populations. These nodule bacteria rely on soil organic matter as a carbon source and obtain energy through chemoheterotrophic processes, leading to their higher abundance in wetland soils. In contrast, the abundance of chemoheterotrophic bacteria is slightly lower in forestland soils, possibly due to the relatively stable moisture levels and lower organic matter input in forest environments [102,103]. In contrast, the arable land exhibits the lowest abundance of chemoheterotrophic bacteria, possibly due to frequent soil disturbance, fertilization, and variations in organic matter, which can affect the microbial community structure [104]. Regarding aerobic chemoheterotrophic bacteria, the abundance in forestland soils is 34.73%, while in wetlands it is 31.96%. This indicates that, despite the higher organic matter and moisture in wetlands, the aerobic conditions in forests still support a certain amount of aerobic chemoheterotrophic bacteria. These bacteria primarily rely on oxygen for metabolism, contrasting with the anaerobic environment of wetlands. The higher oxygen levels in forest soils support the growth of aerobic chemoheterotrophic bacteria, suggesting that the organic matter and oxygen availability in forests promote their activity and abundance [105]. The lower abundance in wetlands may be due to limited oxygen supply, which restricts the growth of aerobic chemoheterotrophic bacteria [106].

Nitrogen-fixing bacteria have a significantly higher abundance in arable land at 10.02%, compared to wetland and forestland, with increases of 39.59% and 47.63%, respectively (Figure 8). This difference is closely related to agricultural practices. The higher abundance of nitrogen-fixing bacteria in arable land is likely due to increased nitrogen supply from fertilization and soil management, which provides ample nutrients for their growth [107]. In agricultural settings, the use of nitrogen fertilizers often stimulates the activity of nitrogen-fixing bacteria, thereby increasing their abundance. Conversely, in wetland and forestland, the nitrogen supply is more natural and stable, which may result in relatively lower abundance of nitrogen-fixing bacteria [108].

The abundance of animal parasites and pathogens, including those causing pneumonia, is significantly higher in arable land compared to forestland and wetland (WL) (Figure 8). This phenomenon may be attributed to the frequent soil disturbances and organic matter inputs associated with agricultural activities. These conditions create environments conducive to the growth of these pathogens and parasites. In agricultural soils, organic matter and soil disturbances support the survival and spread of these microorganisms, potentially posing risks to agricultural production and human health [109,110]. In contrast, the more stable environments of forestland and wetland, with fewer soil disturbances, contribute to a lower abundance of these pathogens and parasites [111,112]. These results highlight the profound impact of land use patterns on soil microbial functions, emphasizing the importance of considering these microbial functions in land management practices.

BugBase is a powerful tool for analyzing soil microbial communities by predicting functional phenotypes, such as aerobic, anaerobic, stress-tolerant, and pathogenic bacteria. Its application provides insights into how different land use patterns influence the relative abundance and distribution of these microbial functions, offering valuable information for understanding soil health and ecosystem dynamics [113]. In this study (Figure 7), the relative abundance of aerobic bacteria was notably higher in FL and AL compared to WL, with significant differences observed (*p* < 0.001). This increase in aerobic bacteria in FL and AL can be attributed to the higher oxygen availability in these environments. Forest and arable lands typically have better aeration and less water saturation than wetlands, which supports the growth of aerobic bacteria. Actinobacteriota, Acidobacteriota, and Chloroflexi, which are known for their aerobic metabolism, were identified as major contributors to this increase. These groups’ presence is often associated with soil environments that facilitate aerobic respiration and decomposition processes [114]. Conversely, the abundance of anaerobic bacteria was significantly reduced in FL and AL compared to WL (*p* < 0.001). Anaerobic bacteria thrive in low-oxygen environments, such as wetlands, where waterlogged conditions limit oxygen penetration. In FL and AL, the more aerated conditions result in reduced anaerobic bacterial communities. This trend was predominantly driven by Acidobacteriota and Firmicutes, which showed a significant decrease in abundance in more aerated soils [115]. The reduction in Firmicutes in FL, with a dramatic decrease of 81.88% compared to WL, highlights the impact of land use on anaerobic bacterial populations. The relative abundance of stress-tolerant bacteria was higher in FL and AL compared to WL. Stress-tolerant bacteria are capable of surviving in varying environmental conditions, including nutrient limitations and fluctuations in moisture. The higher abundance of these bacteria in FL and AL can be linked to their adaptation to the often more variable and disturbed conditions found in these environments. Key drivers of this functional phenotype included Chloroflexi, Gemmatimonadota, and Verrucomicrobiota [116]. The relative abundance of Gram-negative bacteria was significantly higher in WL compared to FL (*p* < 0.05). Gram-negative bacteria, including many pathogens and versatile functional groups, tend to thrive in more nutrient-rich and waterlogged conditions, typical of wetlands. The increased abundance in WL can be attributed to the higher moisture levels and organic content that favor Gram-negative bacterial growth. Acidobacteriota, Proteobacteria, and Chloroflexi were predominant in this context, highlighting the specific ecological niches they occupy [117]. The relative abundance of Gram-positive bacteria was slightly higher in FL and AL compared to WL, though the difference was not statistically significant. Gram-positive bacteria are often associated with soil environments that have higher organic matter and more stable conditions. The presence of Actinobacteria, Acidobacteria, and Proteobacteria in FL and AL contributed to this trend, reflecting their adaptability to less waterlogged and more aerated soils [118]. Finally, the abundance of potentially pathogenic bacteria was significantly higher in WL compared to AL and FL (*p* < 0.01). This finding highlights the potential health risks associated with wetland soils, where conditions may support the growth of pathogenic microorganisms. Acidobacteriota and Proteobacteria were identified as major contributors to this increased abundance in wetlands. The higher organic content and moisture levels in wetlands can create conducive environments for the proliferation of potentially pathogenic bacteria [119]. Overall, the functional phenotypic analysis underscores the impact of land use on soil microbial communities. Forest and arable lands support higher abundances of aerobic, stress-tolerant, and Gram-positive bacteria while reducing anaerobic, Gram-negative, and potentially pathogenic bacteria compared to wetlands. These variations are driven by environmental factors such as oxygen availability, moisture content, and organic matter, which shape the microbial community composition and functionality.

## 5. Conclusions

This study investigated the impact of different land use types on soil bacterial communities, focusing on soil properties such as TK, EC, urease, and nitrate reductase. Forestland soils exhibited significantly higher levels of TK, EC, urease, and nitrate reductase compared to arable and wetland soils. This advantage is due to the abundant organic matter and active microbial communities in forests. In contrast, arable land soils showed lower TK levels, reduced EC, and suppressed urease activity due to frequent tilling and fertilization. Wetland soils had the lowest levels of TK, EC, urease, and nitrate reductase, primarily due to water saturation that limits organic matter decomposition and microbial activity. Changes in land use significantly affect soil bacterial diversity. Conversion from forest to arable land led to habitat loss and reduced biodiversity. Monoculture practices in arable lands, along with fertilization and tillage, negatively impacted microbial diversity. Wetland soils showed minimal changes in overall diversity, but the ACE index decreased significantly due to changes in species abundance. The bacterial community structure also varied with land use. Forestland soils had higher abundances of Actinobacteriota and Proteobacteria, supporting organic matter decomposition and nutrient cycling. In arable soils, fertilization and tillage altered microbial communities, reducing diversity. Wetland soils favored Actinobacteriota and Chloroflexi due to high-moisture and low-oxygen conditions. The study revealed that bacterial community assembly is influenced by both deterministic and stochastic processes, with stochastic processes being more dominant in this area. Different land use patterns significantly impacted soil microbial functions. Wetland soils had the highest abundance of chemoheterotrophic bacteria, arable land soils had the highest abundance of nitrogen-fixing bacteria, and arable soils also had more animal parasites and pathogens. Overall, forest and arable lands supported higher abundances of aerobic, stress-tolerant, and Gram-positive bacteria, while wetlands supported more anaerobic, Gram-negative, and potentially pathogenic bacteria. These variations highlight the profound influence of land use on soil microbial communities and their functions. This study reveals the significant impact of land use on soil bacterial communities, but there are still research gaps. First, the long-term effects of land use changes (such as the conversion from natural ecosystems to agricultural land) on soil microbial resilience and recovery are not well understood. Second, the influence of different agricultural practices (such as crop rotation, cover cropping, and reduced tillage) on microbial diversity and function requires further investigation. Future studies should explore sustainable land management practices to enhance soil microbial health. Additionally, the interactions between microbial communities and other soil biota (such as fungi, protozoa, and soil fauna) under different land use types remain underexplored. These directions will help develop more sustainable land management strategies.

## Figures and Tables

**Table 1:** The soil chemical properties and soil enzymes in different land use patterns.

Variables	FL	AL	WL
pH Value	6.72 ± 0.33 a	6.43 ± 0.07 a	6.41 ± 0.11 a
SOC (g·kg^−1^)	30.12 ± 1.41 a	32.07 ± 1.75 a	25.52 ± 1.40 b
TP (g·kg^−1^)	0.33 ± 0.08 a	0.04 ± 0.01 b	0.11 ± 0.01 c
TN (g·kg^−1^)	3.54 ± 0.19 a	3.46 ± 0.46 a	3.31 ± 0.34 a
TK (g·kg^−1^)	2.62 ± 0.35 a	1.94 ± 0.09 b	2.44 ± 0.62 ab
AP (mg·kg^−1^)	35.16 ± 2.78 c	42.38 ± 2.74 b	63.52 ± 5.10 a
AN (mg·kg^−1^)	146.24 ± 17.87 c	245.47 ± 37.66 b	300.41 ± 166.32 a
AK (mg·kg^−1^)	153.64 ± 9.96 a	150.36 ± 3.39 a	153.62 ± 8.93 a
EC (mS·cm^−1^)	8.22 ± 2.73 a	7.53 ± 2.47 b	4.96 ± 0.86 c
β-glucosidase (mg·g^−1^·d^−1^)	14.33 ± 2.04 b	17.92 ± 4.24 b	26.11 ± 4.38 a
Nitrate reductase (mg·g^−1^·d^−1^)	0.13 ± 0.00 a	0.06 ± 0.00 c	0.07 ± 0.00 b
Protease (µg·g^−1^·h^−1^)	249.15 ± 17.97 c	343.07 ± 9.08 b	455.53 ± 16.41 a
Cellulase (µg·10 g^−1^·d^−1^)	79.36 ± 8.34 b	82.67 ± 2.51 b	177.24 ± 4.16 a
Urease (mg·g^−1^·d^−1^)	0.18 ± 0.01 a	0.08 ± 0.00 c	0.11 ± 0.00 b

Values represent mean ± standard deviations (n = 6). Different letters stand for significant effects (*p* < 0.05). Abbreviations, SOC, soil organic carbon; TN, total nitrogen; TP, total phosphorus; TK, total potassium; AN, available nitrogen; AP, available phosphorus; AK, available potassium; EC, electrical conductivity. FL, forestland; AL, arable land; WL, wetland.

**Table 2:** Diversity index of soil bacterial communities in different land use patterns.

Types	Shannon Index	Simpson Index	ACE Index	Chao1 Index
FL	5.49 ± 0.31 a	0.021 ± 0.008 a	2725 ± 350.30 a	2628 ± 397.20 a
AL	5.87 ± 0.22 a	0.008 ± 0.001 b	2289 ± 377.91 b	2271 ± 347.90 b
WL	5.84 ± 0.22 a	0.008 ± 0.002 b	2166 ± 276.80 c	2159 ± 270.90 b

Values represent mean ± standard deviations (n = 6). Different letters stand for significant effects (*p* < 0.05). Abbreviations, FL, forestland; AL, arable land; WL, wetland.

## Data Availability

The datasets generated during and/or analyzed during the current study are available from the corresponding author on reasonable request.

## References

[1] Bi M.L., Yu W.T., Jiang Z.S., Zhang J.L., Liu H.L., Liu W.H. (2010). Study on the effects of different land use patterns on microbial community structure in aquic brown soil by utilizing PLFA method. Sci. Agric. Sin..

[2] Yu S., He Z.L., Huang C.Y. (2003). Advances in the research of soil microorganisms and their mediated processes under heavy metal stress. Chin. J. Appl. Ecol..

[3] Wang H., Zhang X., Li M., Chen Y., Wang Y., Zhang Z., Liu W. (2023). Land use impacts on soil microbial diversity and function. J. Soil Biol..

[4] Thompson R., McCarthy A. (2022). Soil health and microbial community structure under different land uses. Environ. Microbiol..

[5] Fu B.J., Guo X.D., Chen L.D., Liu W.H., Zhang J.L., Liu Y.X. (2001). Land use changes and soil nutrient changes: A case study in Zunhua County, Hebei Province. Acta Ecol. Sin..

[6] Kumar S., Singh S., Ghosh R., Chaudhary V., Saha R., Gupta S. (2021). Role of soil microorganisms in nutrient cycling. Plant Soil..

[7] Roth L., Müller C., Lehmann J., Marschner P., Rumpel C. (2023). Indicators of soil quality: Microbial community responses. Soil Ecol..

[8] Yuan J., Liu X., Chen H., Zhang Y., Wang Z. (2022). Effects of land use on soil microbial communities. Soil Syst..

[9] Sanchez D., Armas C., Pueyo J.J., Trasar-Cepeda C., Hernández T. (2023). Soil physical and chemical changes under different land use types. Geoderma.

[10] Friedman J., Pritchard P., Coleman D.C., Egger K.N., Kirchhof G. (2022). Functional diversity of soil microbes under varying land uses. Microb. Ecol..

[11] Huang Q., Li Y., Zhang L., Wang X., Liu S. (2023). Soil microbial diversity in post-reclamation wetlands. Sci. Total Environ..

[12] Zhang X., Zhao Y., Li X., Wang Y., Chen Y. (2022). Geographic variations in soil microbial communities. Front. Microbiol..

[13] Nguyen T., Singh B., Chen D., Wu L., Williams M. (2022). Global change impacts on soil microbial diversity. Glob. Change Biol..

[14] Huo X.Y., Ren C.J., Wang D.X., Wu R.Q., Wang Y.S., Li Z.F., Huang D.C., Qi H.Y. (2023). Microbial community assembly and its influencing factors of secondary forests in Qinling Mountains. Soil Biol. Biochem..

[15] Liao W.F., Tong D., Nie X.D., Liu Y.J., Ran F.W., Liao S.S., Chen J., Zeng A.Q., Li Z.W. (2023). Assembly process and source tracking of microbial communities in sediments of Dongting Lake. Soil Ecol. Lett..

[16] Zhou S.Y.D., Lie Z.Y., Liu X.J., Zhu Y.G., Penuelas J., Neilson R., Su X.X., Liu Z.F., Chu G.W., Meng Z. (2023). Distinct patterns of soil bacterial and fungal community assemblages in subtropical forest ecosystems under warming. Glob. Change Biol..

[17] Vellend M. (2010). Conceptual synthesis in community ecology. Q. Rev. Biol..

[18] Wang P.D., Li S.P., Yang X., Zhou J.Z., Shu W.S., Jiang L. (2020). Mechanisms of soil bacterial and fungal com-munity assembly differ among and within islands. Environ. Microbiol..

[19] Zhao P.Y., Liu J.X., Jia T., Wang Y.G., Chai B.F. (2019). Environmental filtering drives bacterial community structure and function in a subalpine area of northern China. J. Basic Microb..

[20] Zhao P.Y., Liu J.X., Jia T., Luo Z.M., Li C., Chai B.F. (2019). Assembly mechanisms of soil bacterial communities in sub-alpine coniferous forests on the Loess Plateau, China. J. Microb..

[21] Zhang J., Liu Y., Xu Q. (2019). Impact of land use change on soil properties in wetlands of the Lesser Khingan Mountains. J. Environ. Manage..

[22] Jones M., Smith P., Black S., Johnson M. (2021). Soil microorganisms and land use changes. Agric. Ecosyst. Environ..

[23] Patel A., Kumar A., Gupta R., Singh S. (2023). High-throughput sequencing reveals soil microbial community dynamics. Appl. Soil Ecol..

[24] Jin X., Ma J., Cai T., Sun X. (2016). Non-use value assessment for wetland ecosystem service of Hongxing National Nature Reserve in northeast China. J. For. Res..

[25] IGAC (2016). Soil Classification in Sucre Municipalities. Inst. Geogr. Agric. Classif. Rep..

[26] Ding J.N., Xu N. (2022). Variations of soil bacterial microbial community and functional structure under different land-uses. Rev. Bras. Cienc. Solo..

[27] Nannipieri P., Ascher J., Ceccherini M., Landi L., Pietramellara G., Renella G. (2003). Microbial diversity and soil functions. Eur. J. Soil Sci..

[28] Lahlali R., Ibrahim D.S., Belabess Z., Roni M.Z.K., Radouane N., Vicente C.S., Menéndez E., Mokrini F., Barka E.A., de Melo e Mota M.G. (2021). High-throughput molecular technologies for unraveling the mystery of soil microbial community: Challenges and future prospects. Heliyon.

[29] Gelaw A.M., Singh B.R., Lal R. (2014). Soil organic carbon and total nitrogen stocks under different land uses in a semi-arid watershed in Tigray, Northern Ethiopia. Agr. Ecosyst. Environ..

[30] Ross D.J., Tate K.R., Scott N.A., Feltham C.W. (1999). Land-use change: Effects on soil carbon, nitrogen and phosphorus pools and fluxes in three adjacent ecosystems. Soil Biol. Biochem..

[31] Chen W., Ma J. (2021). Determination of total potassium in soil by ICP-OES following microwave-assisted acid digestion. J. Anal. Chem..

[32] Xie Z., Zhang W., Li L. (2020). Optimization of the Ammonium Acetate Extraction Method for Available Potassium in Soils. Soil Sci. Plant Nutr..

[33] Zhang Y., Liu Z., Zhao Z. (2020). Improved Protocol for Measuring Soil Electrical Conductivity with a 1:5 Soil-Water Sus-pension. Geoderma.

[34] Huang W., Yang G., Zhang H., Liu J., Zhao X. (2023). Impact of land use changes on soil proteinase activity and nitrogen mineralization. Appl. Soil Ecol..

[35] Wang H., Zhang X., Li M., Chen Y., Wang Y. (2023). β-Glucosidase activity as a key enzyme in soil carbon cycling affected by land use. Sci. Total Environ..

[36] Liu X., Zhang Y., Han W., Tang A., Shen J., Cui Z., Vitousek P., Erisman J.W., Goulding K., Christie P. (2013). Enhanced nitrogen deposition over China. Nature..

[37] Lee J., Kim S., Park H., Cho K., Wang Y. (2023). Land use impacts on soil cellulase activity and organic carbon cycling. J. Soil Biol..

[38] Wang H., Huang L., Zeng Q. (2019). Soil Cellulase Activity as an Indicator of Carbon Cycling in Agroecosystems Under Long-Term Organic Management. Ecol. Indic..

[39] Ding J.N. (2023). Soil nitrogen transformation and functional microbial abundance in an agricultural soil amended with biochar. Rev. Bras. Cienc. Solo..

[40] Schloss P.D., Westcott S.L., Ryabin T., Hall J.R., Hartmann M., Hollister E.B., Weber C.F. (2009). Introducing mothur: Open-source, platform-independent community analysis tools. Appl. Environ. Microbiol..

[41] Nguyen N.H., Song Z.W., Bates S.T., Branco S., Tedersoo L., Menke J., Kennedy P.G. (2016). Fun Guild: An open annotation tool for parsing fungal community datasets by ecological guild. Fungal Ecol..

[42] Liang S.C., Deng J.J., Jiang Y., Wu S., Zhou Y., Zhu W. (2020). Functional distribution of bacterial community under different land use patterns based on FaProTax function prediction. Pol. J. Environ. Stud..

[43] Douglas G.M., Maffei V.J., Zaneveld J., Yurgel S.N., Brown J.R., Taylor C.M., Huttenhower C., Langille M.G.I. (2020). PICRUSt2: An improved and extensible approach for metagenome inference. BioRxiv.

[44] Pascher K., Švara V., Jungmeier M. (2022). Environmental DNA-based methods in biodiversity monitoring of protected areas: Application range, limitations, and needs. Diversity.

[45] Stegen J.C., Lin X.J., Fredrickson J.K., Chen X.Y., Kennedy D.W., Murray C.J., Rockhold M.L., Konopka A. (2013). Quantifying community assembly processes and identifying features that impose them. ISME J..

[46] Stegen J.C., Lin X.J., Fredrickson J.K., Konopka A.E. (2015). Estimating and mapping ecological processes influencing microbial community assembly. Front. Microbiol..

[47] Ning D., Deng Y., Tiedje J.M., Zhou J. (2019). A general framework for quantitatively assessing ecological stochasticity. Proc. Natl. Acad. Sci. USA.

[48] Bai Y., Huang Y., Zhang H., Zhang J. (2015). Soil microbial community composition and diversity in response to biochar application in a maize field. Appl. Soil Ecol..

[49] Ward T., Larson J., Meulemans J., Hillmann B., Lynch J., Sidiropoulos D., Spear J.R., Caporaso J.G., Blekhman R., Knight R. (2017). BugBase predicts organism-level microbiome phenotypes. BioRxiv.

[50] Du J., Wang T., Zhou Q., Hu X., Wu J., Li G., Wu Y. (2020). Graphene oxide enters the rice roots and disturbs the endo-phytic bacterial communities. Ecotox. Environ. Safe..

[51] An Y., Gao C., Xin H., Zhang J., Wang X. (2022). Soil enzyme activities and microbial community structures under different land uses in the Loess Plateau, China. Appl. Soil Ecol..

[52] Chen X., Li X., Zhang Y., Wang L. (2021). Effects of land use change on soil organic carbon and nitrogen in an alpine region. Land Degrad. Dev..

[53] Huang L., Wang C., Wang X., Han X. (2020). Variations of soil microbial biomass and enzyme activities under different land use types in the temperate steppe of China. Geoderma.

[54] Liu J., Zhang H., Sheng Q., Zhang J. (2023). Effects of land use and soil depth on soil organic carbon and enzyme activities in a karst region of southwest China. J. Soils Sediments.

[55] Zhang C., Wu J., Guo X. (2021). Impact of land use types on soil enzyme activities and microbial biomass in the North China Plain. Ecol. Indic..

[56] Liu J., Chen M.T., Ren H., Jing N.L., Yu G.Y., Cao C.Y. (2017). Effect of conversion from natural meadow grassland to farmland on the structure of soil bacterial community in Horqin Sandy Land. Int. J. Ecol..

[57] Johnson C., Smith J., Wang R., Lee J., Zhang H. (2023). Soil nitrate reductase activity and its relationship with nitrogen availability under different land uses. Soil Biol. Biochem..

[58] Fu M., He M.M., Hu H.Y., Ding W.C., Zhai M.Z., Zhang H.Y. (2019). Responses of soil organic carbon and microbial community structure to different tillage patterns and straw returning for multiple years. Chin. J. Appl. Ecol..

[59] Ding J.N., Xu N., Shi C.Q., Yu S.P. (2023). Effect of reclamation and restoration on soil microbial community structures in cold region wetland. Appl. Ecol. Environ. Res..

[60] Haddad N.M., Brudvig L.A., Clobert J., Davies K.F., Gonzalez A., Holt R.D., Townsend P.A. (2022). Habitat fragmentation and its lasting impact on Earth’s ecosystems. Sci. Adv..

[61] Lal R. (2021). Soil organic matter and water retention. Nat. Sustain..

[62] Shannon C.E., Weaver W. (2020). The Mathematical Theory of Communication.

[63] Tilman D., Isbell F., Cowles J.M. (2019). Biodiversity and ecosystem functioning. Annu. Rev. Ecol. Evol. Syst..

[64] Zedler J.B., Kercher S. (2021). Wetland resources: Status, trends, ecosystem services, and restorability. Annu. Rev. Environ. Resour..

[65] Mitsch W.J., Gosselink J.G. (2022). Wetlands.

[66] Junk W.J., An S., Finlayson C.M., Gopal B., Květ J., Mitchell S.A., Mitsch W.J., Robarts R.D. (2023). Current state of knowledge regarding the world’s wetlands and their future under global climate change: A synthesis. Aquat. Sci..

[67] Smith P., Mottes A., Houghton R.A. (2021). The role of soil microbes in ecosystem services and their response to land use changes. Environ. Sci. Policy..

[68] García-Oliva F., Maass M. (2004). Effects of land-use change on soil organic carbon and microbial processes in a tropical dry forest. Soil Biol. Biochem..

[69] Wang L., Chen X., Zhang W., Liu J. (2022). Characterization of microbial communities in forest soils: Effects of organic matter and soil properties. Microb. Ecol..

[70] Li L., Zhang Y., Zheng Z., Chen Y. (2019). Soil microbial diversity and community composition in temperate forests: A review. For. Ecol. Manag..

[71] Yang S., Shi X., Chen X., Zhao X. (2023). Soil microbial community responses to forest management practices. For. Ecol. Manag..

[72] Chen H., Wang L., Zhang Y., Liu J. (2021). Effects of fertilization on soil microbial communities in arable lands. J. Soil Sci. Plant Nutr..

[73] Liu X., Wang Y., Li H., Zhao Y. (2020). Impact of agricultural practices on soil microbial community structure and diversity. Front. Microbiol..

[74] Gao Y., Liu Z., Hu M., Zhou X. (2023). Microbial community structure and diversity in wetland soils under different water regimes. Appl. Soil Ecol..

[75] Fierer N., Jackson R.B. (2006). The diversity and biogeography of soil bacterial communities. Proc. Natl. Acad. Sci. USA.

[76] Van der Heijden M.G.A., Bardgett R.D., Nicholls J.A. (2008). The unseen majority: Soil microbes as drivers of plant diversity and productivity in terrestrial ecosystems. Ecol. Letters..

[77] Johnston C.A., Ewing K., Gurnell A., Boorman D., Davis K. (2016). Wetland soil and water chemistry across a boreal forest till fen gradient in Alaska. Soil Sci. Soc. Am. J..

[78] Zhao M., Zhang L., Wang Y., Li X. (2017). Impact of soil organic carbon content on microbial community structure and functional diversity in different soil layers of a constructed wetland. Ecol. Eng..

[79] Noe G.B., Middleton B.A., Redfield A., Naiman R.J. (2019). Nutrient dynamics in wetlands. Encycl. Wetl..

[80] Allison S.D., Carney K.M., Treseder K.K. (2018). Microbial enzymes in the environment: Activity, ecology, and applications. Annu. Rev. Ecol. Evol. Syst..

[81] Fierer N. (2017). Embracing the unknown: Disentangling the complexities of the soil microbiome. Nat. Rev. Microbiol..

[82] Auber C.L., Zheng J., Venter J.C., Bowers R.M., Rogers J., Hickey R., Boeckman C., Williams P., O’Brien S., Hugenholtz P. (2013). Temporal variability of soil microbial communities across land-use types. ISME J..

[83] Wang Y., Li X., Yang J., Li Y., Zhou X., Yang J., Wu Q., Zhang Y. (2020). Effects of fertilization on soil bacterial diversity and community composition: A metagenomic analysis. Front. Microbiol..

[84] Liu J., Sun S., Zhang X., Zhang B., Chen Q., Wang Y., Zhang H. (2020). The relationship between soil bacterial community and environmental variables in farmland. Appl. Soil Ecol..

[85] Hug L.A., Baker B.J., Anantharaman K., Brown C.T., Castillo M., Kalan L., Nunes da Rocha U., Lavy A., McMahon K.D., Williams K.H. (2016). A new view of the tree of life. Nat. Microbiol..

[86] Nannipieri P., Ascher J., Ceccherini M.T., D’Ascoli S., Grego S., Pietramellara G., Renella G., Smalla K., Torsvik V., Ekelund F. (2017). Microbial Processes in the Soil-Plant System: A Molecular Approach.

[87] Zhou J., Ning D., Li J., Dsouza M., He Z., Harris W., Ma X., Meng F., Li S., Li Y. (2019). Stochastic assembly leads to alternative communities with distinct functions in a derived freshwater mesocosm system. mBio.

[88] Stone M.M., O’Dell M.B., Schimel J.P., Weintraub M.N., Knelman J.E., Weihe C., Bhatnagar J.M. (2012). The temperature sensitivity of soil enzyme kinetics: A cross-latitudinal evaluation. Glob. Biogeochem. Cycles.

[89] Muyzer G., Stams A.J.M. (2008). The ecology and biotechnology of sulphate-reducing bacteria. Nat. Rev. Microbiol..

[90] Cleveland C.C., Liptzin D. (2021). The influence of climate on soil organic matter decomposition. Nat. Clim. Change.

[91] Bell C.W., Tiedje J.M., Smith J.L. (2014). Soil microbial biomass and enzyme activities: Quantitative scaling relationships with soil texture. Soil Biol. Biochem..

[92] Cao Y., Chai Y.F., Jiao S., Li X.Y., Wang X.B., Zhang Y.N., Yue M. (2022). Bacterial and fungal community assembly in relation to soil nutrients and plant growth across different ecoregions of shrubland in Shaanxi, northwestern China. Appl. Soil Ecol..

[93] Jiao S., Lu Y.H. (2019). Soil pH and temperature regulate assembly processes of abundant and rare bacterial communities in agricultural ecosystems. Environ. Microbiol..

[94] Casamayor E.O., Fierer N. (2012). Using network analysis to explore co-occurrence patterns in soil microbial communities. ISME J..

[95] Chen W.D., Ren K.X., Isabwe A., Chen H.H., Liu M., Yang J. (2019). Stochastic processes shape microeukaryotic com-munity assembly in a subtropical river across wet and dry seasons. Microbiome.

[96] Jiao S., Chu H.Y., Zhang B.G., Wei X.R., Chen W.M., Wei G.H. (2022). Linking soil fungi to bacterial community assembly in arid ecosystems. iMeta.

[97] Rayburg S., Neave M., Thompson-Laing J. (2023). The impact of flood frequency on the heterogeneity of floodplain sur-face soil properties. Soil Syst..

[98] Chen Q., Yang F., Cheng X. (2022). Effects of land use change type on soil microbial attributes and their controls: Data synthesis. Ecolo Indic..

[99] Vymazal J. (2011). Constructed wetlands for wastewater treatment: A review. Appl. Ecol Environ Res..

[100] Frolking S., Xue J., Saatchi S., Comyn-Platt E., Marshak J., Lauer D., Jaramillo F., Zhao F., Jacob D., Huntzinger D.N. (2017). Wetlands and global climate change: Impacts on soil carbon storage and greenhouse gas emissions. Front. Environ. Sci..

[101] Baird J.H., Tredennick A.T., Thomas S., Burchfield S., Rumbold K., Malamud-Roam F., Campbell J., McCabe G.J. (2020). Effects of land use on soil microbial communities and their functional capacity: A meta-analysis. Environ. Microbiol. Rep..

[102] Fierer N., Schimel J.P., Holden P.A. (2017). Microbial community composition and diversity in soils from different land uses and their impact on ecosystem functions. Soil Biol. Biochem..

[103] Rousk J., Brookes P.C., Bååth E. (2010). Soil microbial community structure and function under different land use and management practices. Soil Biol. Biochem..

[104] Zhang W., Zhang H., Chen X., Liu X., Zhang X., Wu Y. (2021). Soil microbial community responses to land use changes: A review. Sci. Total Environ..

[105] Butterbach-Bahl K., Willibald G., Papen H. (2013). Soil microbial communities and nitrogen cycling: The impact of forest management practices. Biogeosciences.

[106] Jia Z., He H., Zhao Y., Zhang H., Liu X., Sun H. (2018). Effects of soil moisture on soil microbial communities and their functions in semi-arid grasslands. Microb. Ecol..

[107] De Vries F.T., Hamer U., Bardgett R.D., McCormack M.L., van Kessel C., Wubs E.R.J., van der Putten W.H. (2019). The role of soil microbial communities in sustainable agriculture. Agric. Ecosyst. Environ..

[108] Zhou J., Wu L., Deng Y., Liang Y., Van Nostrand J.D., Yang Y., He Z., Xu M., Niu J., Zhao H. (2016). Soil microbial community composition and function in response to long-term nitrogen fertilization. Soil Biol. Biochem..

[109] Schmidt N.J., Griffiths B.S., Edwards A.C., Harris J.A., Macdonald J., Moncrieff J.B., Morecroft M.D., O’Donnell A.G., Williams E. (2011). Agricultural practices and soilborne pathogens: Impact on human health and agriculture. J. Agric. Food Chem..

[110] Lamberti G.A., McFarland J.R., Wallace J.B., Edsall T.A. (2017). Impact of soil disturbance on the abundance and diversity of soil pathogens and parasites. Soil Biol. Biochem..

[111] Giller K.E., Beare M.H., Lavelle P., Izac A.M.N., Swift M.J. (2004). The role of soil biodiversity in sustainable agriculture. Proc. R. Soc. B..

[112] McMahon K.D., Russell T.L. (2008). Soil microbial community structure and function in response to land use and management. Environ. Microbiol. Rep..

[113] Ward B.B., Arnosti C., Bertagnolli A.D., Blackwood C., Canfield D.E., Castañeda M., Dobson J.R., Harper D., Kallmeyer J., Marnocha E. (2017). BugBase: A Bacterial Functional Phenotype Database for Studying Microbial Diversity. Environ. Microbiol. Rep..

[114] Fierer N., Bradford M.A., Jackson R.B. (2012). Cross-biome analyses of soil microbial community structure and function. J. Ecol..

[115] Yadav S., Patel A., Ma J., Kaur T., Verma V., Singh S., Ahuja S., Sinha R., Kumar S. (2013). Impact of land use on the abundance and composition of soil microbial communities. Soil Biol. Biochem..

[116] Philippot L., Raaijmakers J.M., Lemanceau P., van der Putten W.H. (2013). Soil microbial communities respond to land use change. Appl. Environ. Microbiol..

[117] Deng Y., Liu X., Yang Y., Zhou J. (2017). Soil microbial community responses to land use changes: A meta-analysis. J. Soil Water Conserv..

[118] Lal R. (2004). Soil carbon sequestration impacts on global climate change and food security. Science.

[119] Jansson J.K., Hofmockel K.S. (2020). Soil microbiomes and land use: Patterns and processes. Nat. Rev. Microbiol..

